# Abiotrophia defectiva: A Rare Causative Agent of Infective Endocarditis With Severe Complications

**DOI:** 10.7759/cureus.73715

**Published:** 2024-11-15

**Authors:** Harini Chinnaraj, Maddina Vinay vardhan, Harsha Vardhan Gudibandi, J S Kumar, Subramaniyan Kumarasamy

**Affiliations:** 1 General Medicine, Sri Ramaswamy Memorial (SRM) Medical College Hospital and Research Centre, Chengalpattu, IND

**Keywords:** abiotrophia defectiva, embolization, infective endocarditis, rheumatic heart disease, stroke

## Abstract

Infective endocarditis (IE) primarily affects the endocardium of the heart valves. One of the common neurological complications of IE is the embolization of endocardial vegetation, which can obstruct the cerebral arteries. We describe the case of a 47-year-old male patient who developed neurological symptoms as a complication of IE. *Abiotrophia defectiva*, a nutritionally altered* Streptococcus*, was identified in his blood culture. Treatment of both neurological and vegetative complications is often challenging and necessitates the involvement of a multidisciplinary team. Given the high mortality rate associated with these complications, the diagnosis of IE should be taken into consideration for individuals presenting with fever and neurological abnormalities.

## Introduction

*Abiotrophia defectiva* is a nutritionally modified S*treptococcus*, a normal component of the oral, gastrointestinal, and urogenital tract flora. Endocarditis caused by nutritionally modified* Streptococcus *accounts for approximately 5%-6% of all *streptococcal* endocarditis cases, while <1% of all endocarditis cases are caused by* A. defectiva* [1]. *A. defectiva* is an uncommon but clinically significant cause of infective endocarditis (IE). Its innate resistance to commonly prescribed antibiotics has also been associated with higher rates of morbidity and mortality, underscoring the need for early detection and effective treatment [2]. Additionally, *A. defectiva* is often associated with distal embolization and multiorgan failure.

IE, characterized by an infection within the heart chambers, is a serious condition in which more than 50% of the cases occur in individuals without any underlying cardiac disease [3]. Patients with rheumatic heart disease, valvular abnormalities (such as prostheses, mitral regurgitation, or aortic stenosis), or endocardial injury due to circulating particulate matter as a result of intravenous drug use are at the highest risk for developing endocarditis. The most common extracardiac complications of IE are neurological sequelae, which occur in 25%-50% of patients. IE is twice as prevalent in men and occurs at a rate of 3-9 cases per 100,000 people annually in affluent nations [4]. When endocardial vegetations embolize and block an intracerebral artery, a stroke can occur as a consequence of IE. Compared to people without neurological involvement, those with neurological issues have a higher mortality rate [5]. While *Streptococcus pneumoniae* is the most prevalent organism causing bacterial meningitis as a neurological complication of IE, the isolation of *Staphylococcus aureus* in culture should raise suspicion for foci outside the central nervous system [6]. Due to the indolent course and difficult diagnosis of *A. defectiva* endocarditis, it often leads to a higher mortality rate and severe complications. We report a case of neurological sequelae in IE caused by *A. defectiva *species.

## Case presentation

A 47-year-old male patient presented to the outpatient department with significant clinical symptoms. He reported right-sided weakness in both the upper and lower limbs for the last six hours, accompanied by intermittent high-grade fever for the past four weeks, loss of appetite, and progressive weight loss. Ten days prior, the patient had sought evaluation at an outside hospital for fever and anorexia, where a negative fever panel was obtained (including tests for dengue, microfilaria, microparasites, scrub typhus, and leptospirosis). Blood cultures showed no growth. His medical history included rheumatic heart disease status post-mitral valve replacement and diabetes mellitus. He was taking anticoagulants and oral hypoglycemic agents. The patient denied alcohol use, smoking, or recent intravenous drug use.

**Figure 1:**
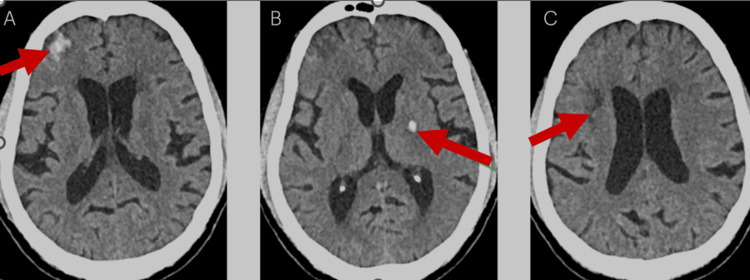
CT of the brain (A) The red arrow highlights the intraparenchymal hemorrhage in the right basifrontal lobe with surrounding edema. (B) The red arrow highlights the hemorrhagic focus in the posterior limb of the left internal capsule. (C) The red arrow highlights a subacute to chronic nonhemorrhagic infarct in the right corona radiata. CT: computed tomography.

On clinical examination, the patient was febrile on arrival but remained conscious, well-oriented, and had stable vital signs. The neurological assessment revealed right-sided paresis affecting both the upper and lower limbs; sensory examination, along with cranial nerve and cerebellum testing, was normal. A cardiovascular exam detected a diastolic murmur in the aortic area and a prosthetic click in the mitral area. Fundoscopic examination revealed Roth’s spots, which are indicative of endocarditis. Neuroimaging via CT of the brain (Figure 1) revealed several significant findings. These included an intraparenchymal hemorrhage in the right basifrontal lobe with surrounding edema and adjacent subarachnoid hemorrhage, a hemorrhagic focus in the posterior limb of the left internal capsule, chronic lacunar infarcts, and a subacute to chronic nonhemorrhagic infarct in the right corona radiata. These findings suggested a combination of embolic events and hemorrhagic complications.

**Figure 2:**
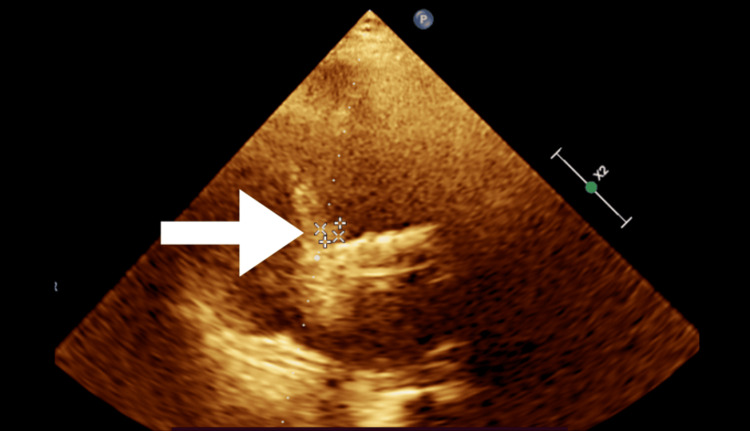
2D echocardiogram image The white arrow highlights the vegetation on the aortic valve.

Laboratory tests showed an international normalized ratio (INR) of 2.9 (therapeutic range, 2.5-3.5), consistent with the patient's mechanical mitral valve replacement and anticoagulation therapy (within target INR). The ESR was elevated, suggesting an inflammatory or infectious process. Blood cultures from three different sites grew *A. defectiva*, a pathogen known to cause IE. A 2D transthoracic echocardiogram (Figure 2; Video 1) revealed vegetation on the aortic valve. Carotid angiography was normal, ruling out carotid artery disease as a source of emboli.

**Video 1:** 2D echocardiogram video of the aortic valve. The white arrow highlights the vegetation in the aortic valve.

A transesophageal echocardiogram (TEE) showed a 9 x 9 mm vegetation on the aortic valve and bileaflet prosthetic valve, with no vegetation in the mitral valve area.

Given the findings, the patient was diagnosed with IE, supported by the presence of vegetation on the aortic valve, blood cultures positive for *A. defectiva*, and Roth’s spots. The neurological symptoms and imaging findings were consistent with embolic stroke complications, resulting in both hemorrhagic and ischemic lesions in the brain.

The patient was promptly started on intravenous antibiotics, including gentamicin 80 mg twice daily and penicillin G six million units every six hours for four weeks, tailored to the sensitivity profile of *A. defectiva*. This was essential for treating the underlying infection. Surgical intervention was performed with an aortic valve replacement to remove the infected valve and prevent further embolic events. Supportive care included careful management of anticoagulation therapy to balance the risk of further bleeding with the need to manage the patient’s underlying condition. Neurological support and rehabilitation were planned to address the residual motor deficits and support recovery. Following the initiation of targeted antibiotic therapy and successful aortic valve replacement, the patient showed marked symptomatic improvement. Regular follow-up was advised to monitor his progress, adjust anticoagulant therapy as needed, and ensure the resolution of the infection. During follow-up, the patient's weakness improved, and he was advised to continue physiotherapy as part of his ongoing rehabilitation.

## Discussion

*A. defectiva*, a nutritionally variant species (NVS), is a facultative anaerobic Gram-positive coccus that was found first in a case of subacute IE. *A. defectiva* is normally found in the oral, gastrointestinal, and urogenital tract flora. NVS as a causative organism of IE accounts for 5%-6% of the cases, and the mortality rate for IE caused by *A. defectiva is *9.2% [1]. The ability of *A. defectiva *to secrete exopolysaccharides and adhere to fibronectin describes its affinity for endovascular tissue, which contributes to the development of complications such as valve destruction, congestive heart failure, vegetation, and septic embolization. The insidious and aggressive course of such vegetations leads to systemic embolization [7]. *A. defectiva* has fastidious growth characteristics, which contribute to negative blood cultures and, consequently, to a delayed and difficult diagnosis. The usual predisposing factors for *A. defectiva* causing IE are prosthetic valves, immunosuppression, pregnancy, poor oral hygiene, and recent dental procedures. Endocarditis caused by *A. defectiva *is characterized by the occurrence of small vegetations and a higher risk of embolisms in one-third of cases, compared to other* streptococci*. The involvement of the aortic valve is more common with this rare infection. *A. defectiva* can also cause osteomyelitis, cerebral abscess, implantable cardioverter defibrillator lead infection, mycotic aneurysm, endophthalmitis, and septic arthritis [8]. As a rare cause of IE, *A. defectiva* more commonly affects the diseased valves, leading to embolic consequences or valvular destruction, compared to normal intact valves [9]. Because of its high resistance to antibiotics and aggressive nature, there is a high possibility of embolic complications and valve destruction, even when treated with antibiotics to which this organism is susceptible. Approximately 50% of patients with *A. defectiva* endocarditis need surgical resection. As discussed earlier, with septic embolization, the mortality rate was higher; therefore, the need for surgical intervention was higher in patients with *A. defectiva*-associated IE than with *Streptococcus*-associated IE [10].

In IE, about 16%-25% of cases present as stroke, with 12%-30% caused by cerebral hemorrhage, as seen in our case. Numerous pathogenic processes contribute to this. Although the exact pathogenesis of cerebral hemorrhage is unknown, hemorrhagic transformation of cerebral ischemic infarcts by septic emboli, rupture of pyogenic arteries, and rupture of mycotic aneurysms are among the plausible explanations [11].

Given the increased risk, critically ill patients with suspected IE should ideally be treated in the intensive care unit to improve outcomes. However, the scheduling of the operation is complicated by neurological problems. The mainstay of medical management is the intravenous infusion of antibiotics according to the sensitivity of the organism [12]. As previously reported, after one week of appropriate treatment, the embolism rate was significantly reduced, and this effect was seen with all of the implicated microorganisms. Tornos et al. observed a strong correlation between the administration of peroral anticoagulant medication at the time of diagnosis and death from neurological impairment in a recent study of 56 individuals with left-sided *S. aureus *IE [13]. Based on this, they advise stopping anticoagulant therapy immediately upon diagnosis and resuming it only after the septic phase of the disease has passed [13].

In cases of heart failure, serious valve damage, or embolic events, a multidisciplinary approach may be necessary in addition to surgery. Studies indicate that 18% of IE cases in Europe involve embolic complications as an indication for surgery, which is performed in about 50% of these cases [14]. Large or mobile vegetations, heart failure, or valvular dysfunction are surgical indications of IE. Surgical treatments for these conditions include valve replacement, repair, or surgical debridement of infected tissue [15].

In patients with IE, neurological consequences have a substantial impact on morbidity and mortality, underscoring the significance of early identification and treatment. A multidisciplinary team, comprising neurologists, infectious disease specialists, cardiologists, cardiothoracic and vascular surgeons, and critical care physicians, is often needed to provide the best possible care [16].

The American Heart Association guidelines recommend dual-agent antibiotic therapy for *A. defectiva*. Use of penicillin G combined with gentamycin for 4-6 weeks is the recommended therapy and may be altered based on culture and sensitivity results [17].

## Conclusions

IE caused by *A. defectiva *is associated with higher mortality and a higher risk of embolization. Hemorrhagic episodes and embolic strokes are two serious systemic consequences that can result from IE. Neurological symptoms should be managed promptly, as they would be in the absence of a septic cause, along with the urgent need to obtain blood cultures and initiate appropriate antibiotic therapy. In our case, the patient presented with neurological complications of IE caused by *A. defectiva* and underwent a surgical procedure after receiving antibiotics tailored to the organism's sensitivity. This case highlights the significance of early detection of common embolism sources, identification of the causative organism, monitoring for complications, and comprehensive examination in individuals presenting with neurological impairments.
